# High-pressure preference for reduced water content in porous zinc aspartate hydrates

**DOI:** 10.1107/S2052520620009348

**Published:** 2020-08-28

**Authors:** Kinga Roszak, Andrzej Katrusiak

**Affiliations:** a Faculty of Chemistry, Adam Mickiewicz University, ul. Uniwersytetu Poznańskiego 8, Poznań 61-614, Poland

**Keywords:** solvates, high pressure, crystallization, polymorphs, phase transitions

## Abstract

The zinc aspartate (ZnAsp_2_) complex, a common dietary supplement, preferentially crystallizes as the dihydrate (ZnAsp_2_·2H_2_O) from aqueous solution. Under normal conditions the dihydrate easily transforms into the sesquihydrate (ZnAsp_2_·1.5H_2_O). The dihydrate crystal structure is triclinic, space group *P* 1, and the sesquihydrate is monoclinic, space group *C*2/*c*. However, their structures are closely related and similarly consist of zinc aspartate ribbons parallel to pores accommodating water molecules. These porous structures can breathe water molecules in and out depending on the temperature and air humidity. High pressure above 50 MPa favours the sesquihydrate, as shown by recrystallizations under pressure and compressibility measured by single-crystal X-ray diffraction up to 4 GPa. This preference is explained by the reduced volume of the sesquihydrate and water compressed separately, compared with the dihydrate. The sesquihydrate undergoes an isostructural phase transition when the voids collapse at 0.8 GPa, whereas no phase transitions occur in the dihydrate, because its pores are supported by increased water content.

## Abstract 2

The zinc aspartate complex, commonly used as a dietary supplement, crystallizes as either the dihydrate or the sesquihydrate. High pressure favours the sesquihydrate, and this has been rationalized by measurements of the compressibility using single-crystal X-ray diffraction up to 4 GPa.

## 1. Introduction   

**Figure 1:**
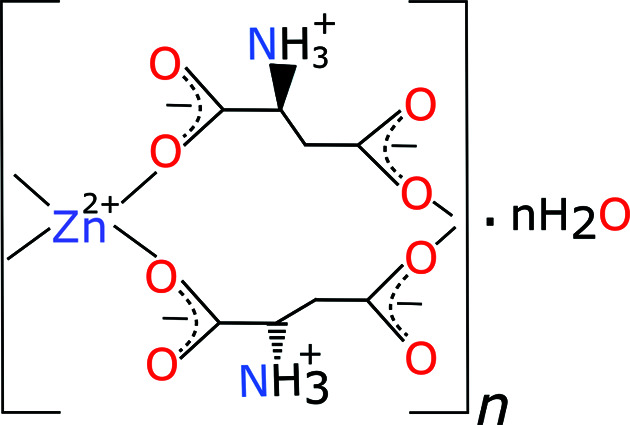
The structural formula of zinc aspartate hydrate, ZnAsp_2_·*n*H_2_O.

Zinc plays a key role in the human body as a cofactor of numerous enzymes and it is therefore often included in dietary supplements and medicines for various illnesses (Shankar & Prasad, 1998 ▸; Haase *et al.*, 2008 ▸; Roohani *et al.*, 2013 ▸; Azeem *et al.*, 2019 ▸). Zinc aspartate hydrate (ZnAsp_2_·*n*H_2_O, Asp denotes the aspartate anion; Fig. 1 ▸) is often used as a source of zinc cations and aspartate amino acids. This racemic complex preferentially crystallizes in the form of hydrates but their structures have not been determined so far.

We have established that crystallization from an aqueous solution under normal conditions yields the dihydrate, ZnAsp_2_·2H_2_O, whereas high-pressure crystallization yields the sesquihydrate, ZnAsp_2_·1.5H_2_O. The structures of both these hydrates are determined here, and the mechanism favouring reduced water content at high pressure is described.

The effect of high pressure on the hydration of various organic compounds, such as methane (Kevenvolden, 1995 ▸), thio­urea (Figuiere *et al.*, 1975 ▸; Tomkowiak *et al.*, 2013 ▸; Tomkowiak & Katrusiak, 2018 ▸), 1,4-di­aza­bicyclo[2.2.2]octane hydro­iodide (dabcoHI, Olejniczak & Katrusiak, 2010 ▸), 1,4-di­aza­bicyclo[2.2.2]octane dibromide (dabco2HBr, Andrzejewski *et al.*, 2011 ▸), 5,6-di­methyl­benzimidazole (Zieliński & Katrusiak, 2015 ▸) and 4,4′-bipyrid­inium perchlorate (Anioła & Katrusiak, 2017 ▸), and minerals (Van Valkenburg *et al.*, 1971 ▸), is well documented in the literature. However, in most cases high pressure increases the water content in a crystal structure, and exceptions are rare. For example, the hydrate Y_2_(C_2_O_4_)_3_·10H_2_O reduces its water content at 1 GPa to Y_2_(C_2_O_4_)_3_·6H_2_O (Zakharov *et al.*, 2017 ▸), the dehydration of phosphatidylinositol bilayers was postulated above 0.7 GPa based on IR spectroscopy (Carrier & Wong, 1996 ▸), and the decomposition of orthoboric acid with the release of water at about 2 GPa was reported by Kuznetsov *et al.* (2006 ▸). We also noted that pressures above 1 GPa prevent the formation of hydrates of thio­urea (Figuiere *et al.*, 1975 ▸; Tomkowiak *et al.*, 2013 ▸; Tomkowiak & Katrusiak, 2018 ▸) and at pressures above 8 GPa no methane hydrates are formed (Kevenvolden, 1995 ▸). Presently, we have established that the porous crystals of ZnAsp_2_·*n*H_2_O display structural features connected to their water content. These features can be conveniently studied and modelled for this compound. We show that sesquihydrate ZnAsp_2_·*n*H_2_O can be employed as an internal component (in tablets) to protect active pharmaceutical ingredients (APIs) from the effects of humidity.

High-pressure studies on APIs increased the number of efficient methods for obtaining new polymorphs (Zakharov *et al.*, 2016*a* ▸,*b* ▸; Neumann *et al.*, 2015 ▸; Boldyreva *et al.*, 2002 ▸; Boldyreva, 2003 ▸; Patyk-Kaźmierczak & Kaźmierczak, 2020 ▸; Fabbiani & Pulham, 2006 ▸; Oswald *et al.*, 2010 ▸) and solvates (Fabbiani *et al.*, 2003 ▸; 2014 ▸). Solvated APIs can be sensitive to the pressure employed in the process of formulation, for example in pressing tablets. High-pressure studies also provide a broader perspective for understanding the thermodynamic transformations of APIs.

## 2. Experimental   

**Figure 2:**
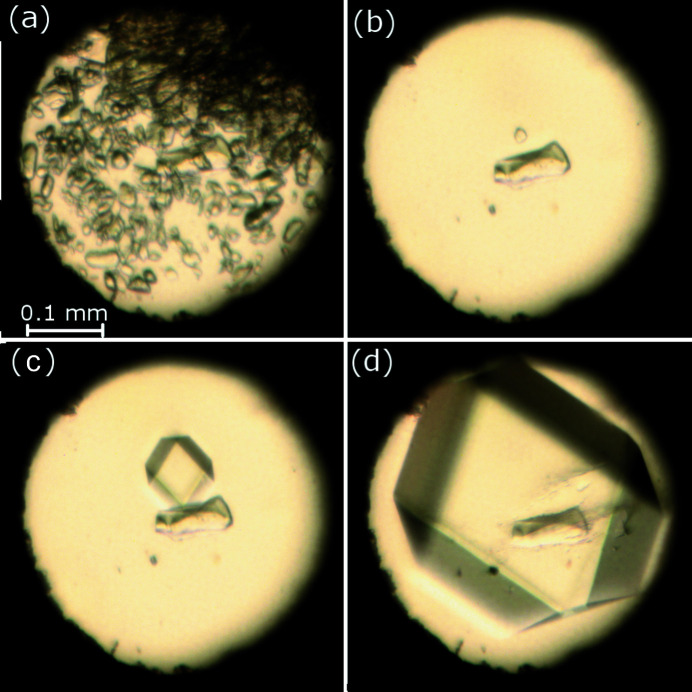
The stages of ZnAsp_2_·1.5H_2_O single-crystal growth from aqueous solution under isochoric conditions in the DAC chamber. (*a*) Spontaneous powder precipitation at 433 K, and one seed at (*b*) 443 K, (*c*) 343 K and (*d*) 0.49 GPa/296 K. The ruby chip for pressure calibration lies near the middle of the chamber.

We performed high-pressure experiments either by gradually compressing a single crystal in a diamond anvil cell (DAC), or by high-pressure recrystallization and growing single crystals in isothermal and isochoric conditions from aqueous solutions *in situ* in the DAC. Experimental parameters and crystal data are listed in Table 1, and in Tables S1 and S2 in the supporting information. High-pressure experiments were performed in a Merrill–Bassett DAC (Merrill & Bassett, 1974 ▸), modified by mounting the anvils directly on steel backing plates with conical windows (Katrusiak, 2008 ▸). The form of the crystals grown was determined by single-crystal X-ray diffraction (Table 1, and Tables S1 and S2 in the supporting information). All isochoric high-pressure recrystallizations yielded the sesquihydrate, ZnAsp_2_·1.5H_2_O. Its growth process is illustrated in Fig. 2 ▸ (see also Fig. S1 in the supporting information).

The sesquihydrate could be recovered from the DAC and no visible changes occurred to the samples exposed to air for weeks. On the other hand, the initially crystallized dihydrate powder kept in a thermally closed plastic bag for two months fully transformed into the sesquihydrate. This indicates that the dihydrate is stable only when submerged in water and in highly humid environments.

The compression of ZnAsp_2_·1.5H_2_O and ZnAsp_2_·2H_2_O crystals was determined for the samples mounted in the DAC. The gasket was made of 0.2 mm thick Inconel foil and the initial diameter of the spark-eroded hole was 0.45 mm. Glycerine was used as a hydro­static medium. The pressure in the DAC was calibrated before and after each diffraction measurement by the ruby fluorescence method (Piermarini *et al.*, 1975 ▸; Mao *et al.*, 1986 ▸) using a Photon Control Inc. spectrometer of enhanced resolution, affording an accuracy of 0.02 GPa.

Single-crystal high-pressure data were measured on a KUMA KM4 CCD diffractometer according to the procedure described previously (Budzianowski & Katrusiak, 2004 ▸). The *CrysAlisPro* software (Rigaku Oxford Diffraction, 2015 ▸) was used for diffraction data collection and preliminary reduction. Reflections that overlapped with diamond reflections were eliminated, and corrections for the DAC and sample absorption and for beam shadowing by the gasket were applied. Using *OLEX2* (Dolomanov *et al.*, 2009 ▸), the crystal structures were solved by intrinsic phasing with *SHELXT* (Sheldrick, 2015*a* ▸) and refined by least-squares with the program *SHELXL* (Sheldrick, 2015*b* ▸). The ambient-pressure structures were used as the starting models for refinement of the high-pressure data. Anisotropic displacement factors were generally applied for non-hydrogen atoms. C- and N-bound H atoms were located from the molecular geometry (assuming distances of C—H = 0.97 Å for methyl­ene, C—H = 0.98 Å for methine and N—H = 0.89 Å for NH_3_). The water H atoms were located from difference Fourier maps and then the positions of H_2_O were refined as rigid units. The *U*_iso_ values of the H atoms were constrained to 1.2 times the *U*_eq_ of their carrier atoms. Structural drawings were prepared using the program *Mercury CSD 3.3* (Macrae *et al.*, 2020 ▸). A total of 26 different data sets were collected, at 15 different pressures for the sesquihydrate and 11 different pressures for the dihydrate. The crystal data have been deposited in the Cambridge Structural Database (CSD; Groom *et al.*, 2016 ▸) as supplementary publications (CSD 1972145–1972170).

Powder X-ray diffraction (XRPD) patterns were recorded using a Bruker AXS D8 Advance diffractometer, equipped with a sealed X-ray tube, a Johansson monochromator selecting Cu *K*α_1_ radiation (λ = 1.54060 Å) and a LynxEye detector. The samples were gently pressed into a flat round cuvette of about 0.4 cm^3^ in volume. The cuvette was rotated during the measurements, performed in θ steps of 0.02° and in the θ–2θ mode.

Thermogravimetric (TG) and differential scanning calorimetry (DSC) analyses were performed for 18.2 mg of ZnAsp_2_·2H_2_O in an N_2_ atmosphere on a Setsys 1200 Setaram instrument between 293 K and 573 K at a scan speed of 5 K min^−1^.

## 3. Results and discussion   

**Figure 3:**
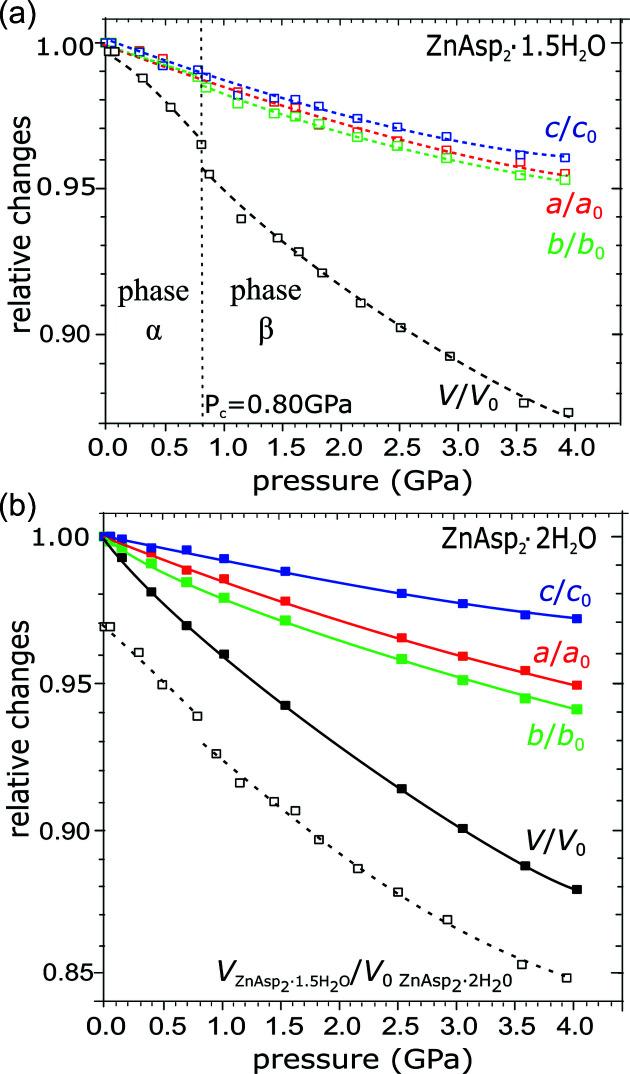
Unit-cell dimensions of (*a*) ZnAsp_2_·1.5H_2_O (open symbols, dashed lines) and (*b*) ZnAsp_2_·2H_2_O (solid symbols and lines), relative to their values at 296 K/0.1 MPa, as a function of pressure. The magnitudes of the unit-cell parameters, including the angular dimensions, are plotted in Figs. S2 and S3. The vertical dashed line in panel (*a*) indicates the phase transition in ZnAsp_2_·1.5H_2_O (see also Fig. S6). The open black symbols in panel (*b*) show the volume compression in ZnAsp_2_·1.5H_2_O relative to the volume of ZnAsp_2_·2H_2_O at 0.1 MPa.

The compressed crystals of ZnAsp_2_·1.5H_2_O and ZnAsp_2_·2H_2_O retain the ambient-pressure monoclinic symmetry of space group *C*2/*c* and triclinic symmetry of space group 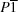, respectively, up to 4 GPa at least (Table 1 ▸, Fig. 3 ▸, Figs. S2 and S3).

Although seemingly very different (Table 1 ▸), the crystal structures of ZnAsp_2_ dihydrate and sesquihydrate are strikingly similar. Their Bravais lattices can be transformed into one another through the following matrix **M**:
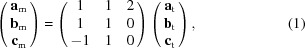
where the subscripts m and t refer to the (pseudo)monoclinic *C* lattice of (ZnAsp_2_·1.5H_2_O) and the triclinic *P* lattice of (ZnAsp_2_·2H_2_O), respectively. The reverse matrix **M** ^−1^ transforms the lattice *C*_m_ into *P*_t_:
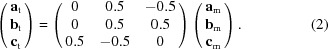
Thus the triclinic *P* unit cell of ZnAsp_2_·2H_2_O at 0.1 MPa can be represented as the pseudo-monoclinic unit cell *C*, having the following dimensions: *a* = 16.445 Å, *b* = 10.804 Å, *c* = 14.780 Å, α = 85.28°, β = 92.27°, γ = 89.66°, which are similar to the unit cell of ZnAsp_2_·1.5H_2_O (Table 1 ▸). According to the unit-cell angles of the pseudo-monoclinic *C* lattice of ZnAsp_2_·2H_2_O (Fig. S3), with increasing pressure this triclinic structure only hardly, within about 0.5°, changes its distortions from the monoclinic symmetry.

Thus the sorption of water molecules into the ZnAsp_2_·*n*H_2_O framework results in its transformation, changing the symmetry of the crystal structure. It is characteristic that the monoclinic symmetry of space group *C*2/*c* of ZnAsp_2_·1.5H_2_O is reduced to the triclinic space group 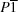 of ZnAsp_2_·2H_2_O after the water content increases. This inverse sorption–symmetry relation is surprising, as according to our survey an increased water content of the pores either increases or preserves the crystal symmetry. For example, the space-group symmetry *R*3 of anhydrate stepanovite polymorph ST1d increases to space group *R*3*c* for stepanovite [Mg(H_2_O)_6_][NaFe(C_2_O_4_)_3_]·3H_2_O; for the polymorph ST2d its anhydrate has space-group symmetry *P*3 and after hydration the symmetry increases to *P*3*c* (Huskić *et al.*, 2019 ▸); the space-group symmetries *P*2_1_/*n* and 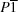 of lithium acetate (LiC_2_H_3_O_2_) polymorphs increase to *Cmmm* for the dihydrate; and space group 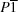 of lithium acetate monohydrate, 4(LiC_2_H_3_O_2_)·H_2_O, increases to *P*2_1_/*c* for the tetrahydrate 4(LiC_2_H_3_O_2_)·4H_2_O (Martínez-Casado *et al.*, 2011 ▸).

**Figure 4:**
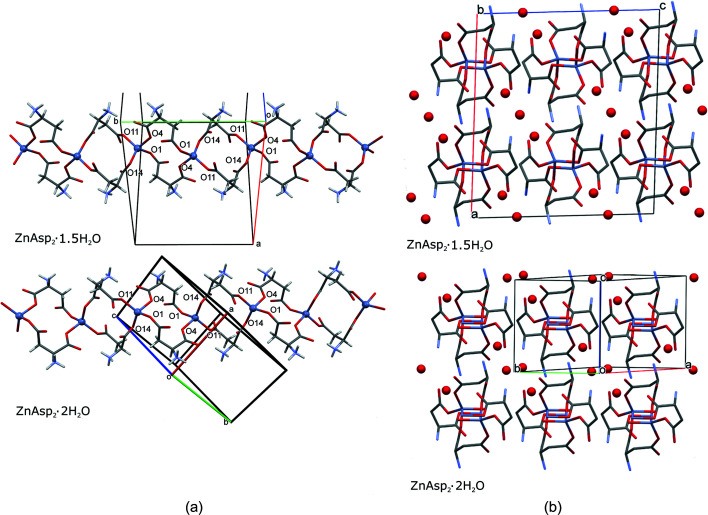
(*a*) Autostereographic projections (Katrusiak, 2001 ▸) of the ribbons formed by the Zn cations four-fold coordinated by Asp anions in ZnAsp_2_·1.5H_2_O and ZnAsp_2_·2H_2_O. Water molecules have been omitted for clarity. (*b*) The ZnAsp_2_·1.5H_2_O and ZnAsp_2_·2H_2_O structures projected down the ZnAsp_2_ ribbons. Water molecules are represented as red balls. H atoms have been omitted for clarity.

We have established that ZnAsp_2_ preferentially crystallizes as the dihydrate when crystallized from water solution under normal conditions (298 K, 0.1 MPa). The dihydrate crystals are triclinic (Table 1 ▸). In these crystal structures the water molecules are located in channel pores and do not participate in the Zn^2+^ coordination. In ZnAsp_2_·2H_2_O the Zn^2+^ cation is coordinated by four carboxyl­ate oxygens of four Asp anions, as illustrated in Fig. 4 ▸. Each Asp participates in coordinating two Zn^2+^ cations, closing cyclamers of the form –Zn^2+^–Asp–Zn^2+^–Asp–, and these are combined into chains extending along the [010] crystal direction. In ZnAsp_2_·1.5H_2_O the Zn^2+^ cation is coordinated in the same way as in ZnAsp_2_·2H_2_O. The ZnAsp_2_ ribbons are very similar in the dihydrate and sesquihydrate, as shown in Fig. 4 ▸. The conformation of the Asp units and their coordination of Zn^2+^ is consistent to within a few degrees for the corresponding torsion angles (Fig. S4).

It is a common feature of both ZnAsp_2_·1.5H_2_O and ZnAsp_2_·2H_2_O that all water molecules interact through O—H⋯O and N—H⋯O hydrogen bonds, but no water mol­ecules participate in Zn coordination. There are very similar intramolecular N13—H13*C*⋯O11 and N3—H3*A*⋯O2 hydrogen bonds in both ZnAsp_2_·1.5H_2_O and ZnAsp_2_·2H_2_O. Almost all of the hydrogen bonds involve water molecules. The hydrogen bonds to H_2_O molecules are O1*W*—H1*WB*⋯O14, O1*W*—H1*WA*⋯O2, O2*W*—H2*WB*⋯O12 and N3—H3*C*⋯O1*W*. Two other hydrogen bonds are N13—H13*B*⋯O2 and N3—H3*B*⋯O13, both present in the ZnAsp_2_·1.5H_2_O and ZnAsp_2_·2H_2_O structures. The O2*W*—H2*WA*⋯O1*W* bond between two water molecules is formed only in ZnAsp_2_·2H_2_O (Figs. S5 and S9).

**Figure 5:**
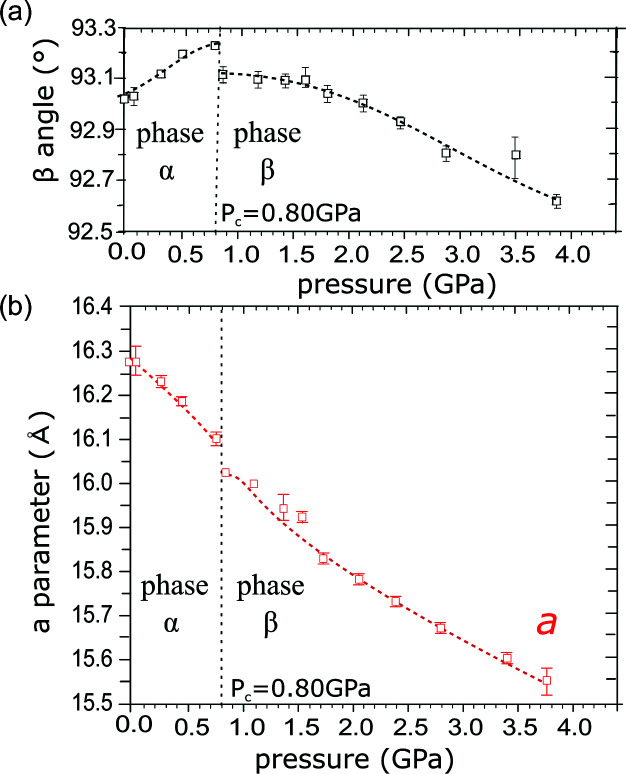
The pressure dependence of (*a*) angle β and (*b*) parameter *a* of the unit cell in ZnAsp_2_·1.5H_2_O. The changes in all the unit-cell dimensions are plotted in Fig. S2.

**Figure 6:**
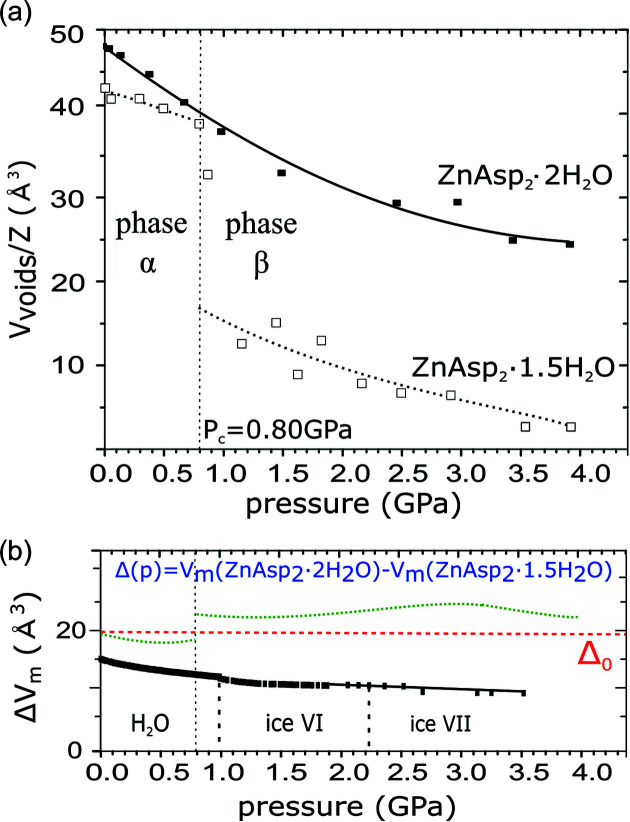
(*a*) The pressure dependence of the void volume (*V*_voids_) of ZnAsp_2_·1.5H_2_O (open symbols, dotted lines) and ZnAsp_2_·2H_2_O (solid symbols, continuous line) after removing the water molecules from these structures, per one formula unit (*V*_voids_/*Z*). The void volume was calculated using the program *Mercury* (Macrae *et al.*, 2020 ▸) with a probing-sphere radius of 1.2 Å and 0.1 Å steps. (*b*) The molecular volume of water (*i.e.* one H_2_O molecule in liquid; Bridgman, 1935 ▸) and ices VI (Kuhs *et al.*, 1984 ▸) and VII (Bezacier *et al.*, 2014 ▸) as a function of pressure compared with the difference in molecular volume (*V*_m_ = *V*/*Z*) between ZnAsp_2_·2H_2_O and ZnAsp_2_·1.5H_2_O (Δ*V* *m*). Δ_0_ is this difference at 0.1 MPa (see also Fig. S6).

It is remarkable that although ZnAsp_2_·2H_2_O and ZnAsp_2_·1.5H_2_O can interconvert one into the other under ambient conditions, each of them can be compressed in glycerine to 4 GPa at least (Fig. 3 ▸). However, while the compression of ZnAsp_2_·2H_2_O is monotonic, in ZnAsp_2_·1.5H_2_O we have noted an anomalous strain at about 0.8 GPa. This anomaly is clearly seen in the pressure dependence of the monoclinic angle β, which initially rises and at 0.8 GPa drops abruptly by 0.1°, and then it continues to drop in a monotonous way it at still higher pressure (Fig. 5 ▸). A similar discontinuity is observed in the compression of unit-cell parameter *a* [Fig. 5 ▸(*b*)] and in the volume compression (Fig. 3 ▸). It appears that the anomalous ‘compression’ of the β angle in ZnAsp_2_·1.5H_2_O is a consequence of the directional interactions – Zn—O coordination and O—H⋯O hydrogen bonds – forming the framework in this structure. These directional bonds play a dominant role in the dimensions and elastic properties of the crystal. Owing to this relatively rigid framework, some small voids are present in the crystal under normal conditions. However, above 0.8 GPa the directional bonds yield under the external pressure and the small voids are suppressed. Thus the anomalous compression marks the pressure value at which central forces supported by the external pressure overcome the angular dimensions favoured by the directional interactions, triggering a collapse to a more densely packed structure [Fig. 6 ▸(*a*)]. These two phases of ZnAsp_2_·1.5H_2_O will be further referred to as phases α and β.

The ZnAsp_2_·1.5H_2_O structure determined at 0.9 GPa is an average of these phases, as the X-ray diffraction measurement was performed when the sample had partly transformed between phases α and β. The anomalous compression at 0.8 GPa, determined from the unit-cell dimensions, agrees well with the collapse of the pores. Their volume is plotted as a function of pressure in Fig. 6 ▸ (note the ‘intermediate’ volume of the pores at 0.9 GPa, due to the averaged structures of phases α and β, as explained above). According to the X-ray diffraction data, the transition at 0.8 GPa does not change the crystal symmetry. Thus it can be classified as an isostructural phase transition, which is quite common for metal–organic frameworks under pressure. For example, at 0.40 GPa Cd(APP)_2_NO_3_·NO_3_ transforms between monoclinic phases, both of space-group symmetry *P*2_1_/*c* (Półrolniczak *et al.*, 2018 ▸) and Co_2_(4,4′-bpy)_3_(NO_3_)_4_·*x*H_2_O preserves its orthorhombic space group *Ccca* at *p*_c_ = 6 GPa (Zhou *et al.*, 2014 ▸), plus other known examples (Moggach *et al.*, 2009 ▸; McKellar & Moggach, 2015 ▸; Sobczak *et al.*, 2018 ▸; Bhattacharyya *et al.*, 2019 ▸).

Interestingly, the subtraction of the molecular volume of ZnAsp_2_·1.5H_2_O from that of ZnAsp_2_·2H_2_O (denoted Δ*V*_m_) should give the volume difference corresponding to half of the molecular volume of water [Fig. 6 ▸(*b*)]. The comparison of Δ*V*_m_ with the volume of one H_2_O molecule in water and ices shows that there is more space in the pores than required for the accommodated water molecules, which explains the reason for the collapse of the pores. The work contribution to the Gibbs free energy at the transition in ZnAsp_2_·1.5H_2_O is 3.1 kJ mol^−1^, compared with the work energy of 4.9 kJ mol^−1^ performed by the pressure to compress phase α from 0.1 MPa to 0.8 GPa. The volume difference between ZnAsp_2_·2H_2_O and ZnAsp_2_·1.5H_2_O at 0.1 MPa is consistent with the broad distribution of the volume of solvent water occurring in organic pharmaceuticals (Glasser, 2019 ▸).

## 4. Practical implications   

**Figure 7:**
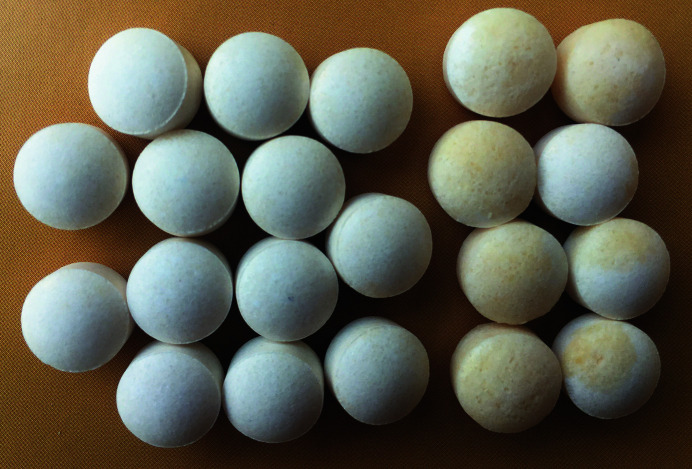
Tablets containing ZnAsp_2_·2H_2_O, some of which show clear signs of the release of water on the spontaneous transformation to ZnAsp_2_·1.5H_2_O: in the eight tablets grouped on the right-hand side of the photograph the degradation is visible as a beige shade and swelling, which leads to their easy abrasion and crushing.

The subtle balance of water content in these two hydrates of zinc aspartate (ZnAsp_2_) has implications for its use as a pharmaceutical agent. The undesired release of water in tablets results in their degradation, as illustrated in the photograph in Fig. 7 ▸. These undesired effects can be avoided by using zinc aspartate in the form of the sesquihydrate, ZnAsp_2_·1.5H_2_O, which is more stable and does not release or absorb water. The sesquihydrate can be obtained either by drying the dihydrate precipitate obtained from aqueous solution, or by performing the crystallization under high-pressure conditions. The high pressure of about 50 MPa required for this purpose is technologically attainable for larger amounts of the sample, although it would require some safety measures. High-pressure crystallization could be more advantageous in terms of the time needed to obtain the required product. Thus the high-pressure crystallization of zinc aspartate can be regarded as a potential candidate for pharmaceutical applications of high pressure (see also Fabbiani *et al.*, 2014 ▸).

## 5. Conclusions   

We have established that the sesquihydrate ZnAsp_2_·1.5H_2_O is a stable form of the zinc aspartate complex (ZnAsp_2_) under ambient conditions. We have shown that the precipitate obtained by crystallization from aqueous solution is the dihydrate, ZnAsp_2_·2H_2_O. The crystal structures of the sesquihydrate ZnAsp_2_·1.5H_2_O and the dihydrate ZnAsp_2_·2H_2_O are closely related, despite their different space-group symmetries.

The most eminent feature of these structures is that they form frameworks containing channel pores that are occupied by water molecules. The water molecules can enter the pores of the sesquihydrate ZnAsp_2_·1.5H_2_O when it is immersed in water, or leave the pores when the dihydrate ZnAsp_2_·2H_2_O is exposed to the air. This transformation of the dihydrate into the sesquihydrate is a slow process, taking days or weeks depending on the volume of the sample, its container, the size of the crystal grains, the humidity of the air, ventilation, temperature and other relevant parameters. Thus it is recommended to check whether the transformation into the sesquihydrate has been completed before further processing, for example granulating or tabletting.

The sesquihydrate can also be obtained directly by high-pressure crystallization. We found that an applied pressure as low as 50 MPa favours crystallization of the sesquihydrate from aqueous solution at 298 K.

The subtle isostructural phase transition in ZnAsp_2_·1.5H_2_O illustrates the role of directional interactions in stabilizing porous structures. It can be postulated that the sesquihydrate is favoured by high pressure because it increases the close packing of molecules in the structure and destabilizes the directional interactions, like hydrogen bonds, which bind the water molecules into the pores. However, it is apparent from other reverse effects on other hydrates that the pressure effects are quite complex and depend on specific structural features.

It should be noted that all our attempts to recrystallize racemic ZnAsp_2_ hydrates never resulted in the separation of enantiomers. This negative result is consistent with the effect of pressure on other racemic mixtures of enantiomers reported so far (*e.g.* Jacques *et al.*, 1994 ▸; Rietveld *et al.*, 2011 ▸; Cai *et al.*, 2013 ▸; Marciniak *et al.*, 2014 ▸; Ostrowska *et al.*, 2015 ▸; Wang *et al.*, 2015 ▸; Ernst, 2018 ▸; Hochberg & Cintas, 2018 ▸; Roszak & Katrusiak, 2018 ▸).

## Supplementary Material

Crystal structure: contains datablock(s) global, Zn-aspartate_sesquihydrate_0_001GPa, Zn-aspartate_sesquihydrate_0_05GPa, Zn-aspartate_sesquihydrate_0_29GPa, Zn-aspartate_sesquihydrate_0_49GPa, Zn-aspartate_sesquihydrate_0_79GPa, Zn-aspartate_sesquihydrate_0_85GPa, Zn-aspartate_sesquihydrate_1_15GPa, Zn-aspartate_sesquihydrate_1_44GPa, Zn-aspartate_sesquihydrate_1_62GPa, Zn-aspartate_sesquihydrate_1_82GPa, Zn-aspartate_sesquihydrate_2_15GPa, Zn-aspartate_sesquihydrate_2_49GPa, Zn-aspartate_sesquihydrate_2_91GPa, Zn-aspartate_sesquihydrate_3_54GPa, Zn-aspartate_sesquihydrate_3_92GPa, Zn-aspartate_dihydrate_0_001GPa, Zn-aspartate_dihydrate_0_05GPa, Zn-aspartate_dihydrate_0_15GPa, Zn-aspartate_dihydrate_0_40GPa, Zn-aspartate_dihydrate_0_70GPa, Zn-aspartate_dihydrate_1_02GPa, Zn-aspartate_dihydrate_1_54GPa, Zn-aspartate_dihydrate_2_53GPa, Zn-aspartate_dihydrate_3_05GPa, Zn-aspartate_dihydrate_3_53GPa, Zn-aspartate_dihydrate_4_02GPa. DOI: 10.1107/S2052520620009348/lo5070sup1.cif

Structure factors: contains datablock(s) zinc-aspartate_sesquihydrate_0_001GPa. DOI: 10.1107/S2052520620009348/lo5070zinc-aspartate_sesquihydrate_0_001GPasup2.hkl

Structure factors: contains datablock(s) zinc-aspartate_sesquihydrate_0_05GPa. DOI: 10.1107/S2052520620009348/lo5070zinc-aspartate_sesquihydrate_0_05GPasup3.hkl

Structure factors: contains datablock(s) zinc-aspartate_sesquihydrate_0_29GPa. DOI: 10.1107/S2052520620009348/lo5070zinc-aspartate_sesquihydrate_0_29GPasup4.hkl

Structure factors: contains datablock(s) zinc-aspartate_sesquihydrate_0_49GPa. DOI: 10.1107/S2052520620009348/lo5070zinc-aspartate_sesquihydrate_0_49GPasup5.hkl

Structure factors: contains datablock(s) zinc-aspartate_sesquihydrate_0_79GPa. DOI: 10.1107/S2052520620009348/lo5070zinc-aspartate_sesquihydrate_0_79GPasup6.hkl

Structure factors: contains datablock(s) zinc-aspartate_sesquihydrate_0_85GPa. DOI: 10.1107/S2052520620009348/lo5070zinc-aspartate_sesquihydrate_0_85GPasup7.hkl

Structure factors: contains datablock(s) zinc-aspartate_sesquihydrate_1_15GPa. DOI: 10.1107/S2052520620009348/lo5070zinc-aspartate_sesquihydrate_1_15GPasup8.hkl

Structure factors: contains datablock(s) zinc-aspartate_sesquihydrate_1_44GPa. DOI: 10.1107/S2052520620009348/lo5070zinc-aspartate_sesquihydrate_1_44GPasup9.hkl

Structure factors: contains datablock(s) zinc-aspartate_sesquihydrate_1_62GPa. DOI: 10.1107/S2052520620009348/lo5070zinc-aspartate_sesquihydrate_1_62GPasup10.hkl

Structure factors: contains datablock(s) zinc-aspartate_sesquihydrate_1_82GPa. DOI: 10.1107/S2052520620009348/lo5070zinc-aspartate_sesquihydrate_1_82GPasup11.hkl

Structure factors: contains datablock(s) zinc-aspartate_sesquihydrate_2_15GPa. DOI: 10.1107/S2052520620009348/lo5070zinc-aspartate_sesquihydrate_2_15GPasup12.hkl

Structure factors: contains datablock(s) zinc-aspartate_sesquihydrate_2_49GPa. DOI: 10.1107/S2052520620009348/lo5070zinc-aspartate_sesquihydrate_2_49GPasup13.hkl

Structure factors: contains datablock(s) zinc-aspartate_sesquihydrate_2_91GPa. DOI: 10.1107/S2052520620009348/lo5070zinc-aspartate_sesquihydrate_2_91GPasup14.hkl

Structure factors: contains datablock(s) zinc-aspartate_sesquihydrate_3_54GPa. DOI: 10.1107/S2052520620009348/lo5070zinc-aspartate_sesquihydrate_3_54GPasup15.hkl

Structure factors: contains datablock(s) zinc-aspartate_sesquihydrate_3_92GPa. DOI: 10.1107/S2052520620009348/lo5070zinc-aspartate_sesquihydrate_3_92GPasup16.hkl

Structure factors: contains datablock(s) zinc-aspartate_dihydrate_0_001GPa. DOI: 10.1107/S2052520620009348/lo5070zinc-aspartate_dihydrate_0_001GPasup17.hkl

Structure factors: contains datablock(s) zinc-aspartate_dihydrate_0_05GPa. DOI: 10.1107/S2052520620009348/lo5070zinc-aspartate_dihydrate_0_05GPasup18.hkl

Structure factors: contains datablock(s) zinc-aspartate_dihydrate_0_15GPa. DOI: 10.1107/S2052520620009348/lo5070zinc-aspartate_dihydrate_0_15GPasup19.hkl

Structure factors: contains datablock(s) zinc-aspartate_dihydrate_0_40GPa. DOI: 10.1107/S2052520620009348/lo5070zinc-aspartate_dihydrate_0_40GPasup20.hkl

Structure factors: contains datablock(s) zinc-aspartate_dihydrate_0_70GPa. DOI: 10.1107/S2052520620009348/lo5070zinc-aspartate_dihydrate_0_70GPasup21.hkl

Structure factors: contains datablock(s) zinc-aspartate_dihydrate_1_02GPa. DOI: 10.1107/S2052520620009348/lo5070zinc-aspartate_dihydrate_1_02GPasup22.hkl

Structure factors: contains datablock(s) zinc-aspartate_dihydrate_1_54GPa. DOI: 10.1107/S2052520620009348/lo5070zinc-aspartate_dihydrate_1_54GPasup23.hkl

Structure factors: contains datablock(s) zinc-aspartate_dihydrate_2_53GPa. DOI: 10.1107/S2052520620009348/lo5070zinc-aspartate_dihydrate_2_53GPasup24.hkl

Structure factors: contains datablock(s) zinc-aspartate_dihydrate_3_05GPa. DOI: 10.1107/S2052520620009348/lo5070zinc-aspartate_dihydrate_3_05GPasup25.hkl

Structure factors: contains datablock(s) zinc-aspartate_dihydrate_3_53GPa. DOI: 10.1107/S2052520620009348/lo5070zinc-aspartate_dihydrate_3_53GPasup26.hkl

Structure factors: contains datablock(s) zinc-aspartate_dihydrate_4_02GPa. DOI: 10.1107/S2052520620009348/lo5070zinc-aspartate_dihydrate_4_02GPasup27.hkl

Additional tables and figures. DOI: 10.1107/S2052520620009348/lo5070sup28.pdf

CCDC references: 1972145, 1972146, 1972147, 1972148, 1972149, 1972150, 1972151, 1972152, 1972153, 1972154, 1972155, 1972156, 1972157, 1972158, 1972159, 1972160, 1972161, 1972162, 1972163, 1972164, 1972165, 1972166, 1972167, 1972168, 1972169, 1972170

## Figures and Tables

**Table 1:** Selected crystal data for ZnAsp_2_·1.5H_2_O and ZnAsp_2_·2H_2_O, all at 296 K (see also Tables S1 and S2 in the supporting information)

	ZnAsp_2_·1.5H_2_O		ZnAsp_2_·2H_2_O	
Pressure (GPa)	0.0001	3.92	0.0001	4.02
Crystal system	Monoclinic	Monoclinic	Triclinic	Triclinic
Space group	*C*2/*c*	*C*2/*c*	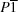	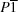
*a* (Å)	16.2789 (3)	15.550 (3)	8.7873 (3)	8.343 (7)
*b* (Å)	10.7307 (2)	10.2262 (7)	9.5061 (3)	8.947 (6)
*c* (Å)	14.5393 (3)	13.966 (1)	9.8114 (3)	9.537 (3)
α (°)	90	90	111.784 (3)	110.02 (4)
β (°)	93.017 (2)	92.62 (3)	105.625 (3)	104.76 (4)
γ (°)	90	90	107.721 (3)	109.16 (7)
*V* (Å^3^)	2536.26 (8)	2219 (4)	653.73 (4)	575.2 (7)
*Z*/*Z*′	4/0.5	4/0.5	1/0.5	1/0.5
*D_x_* (g cm^−3^)	1.868	2.135	1.857	2.111
