# Supplementary material for: High-pressure preference for reduced water content in porous zinc aspartate hydrates
Source: Acta Crystallogr B Struct Sci Cryst Eng Mater. 2020 Aug 28;76(Pt 5):795–801. doi: 10.1107/S2052520620009348 (PMC7535066; doi:10.1107/S2052520620009348)
Supplement: Supplementary file 28 [file b-76-00795-sup28.pdf]

STRUCTURAL SCIENCE
CRYSTAL ENGINEERING
MATERIALS

Volume 76 (2020)

Supporting information for article:

High-pressure preference for reduced water content in porous zinc aspartate hydrates

Kinga Roszak and Andrzej Katrusiak

S1. Tables

S1.1. Crystallographic details

Table S1 Crystallographic data of $ZnAsp_2 \cdot 1.5H_2O$ and experimental details.

Pressure (GPa)		0.001	0.05	0.29	0.49	0.79
Formula weight		713.18	713.18	713.18	713.18	713.18
Wavelength (Å)		1.54184	0.71073	0.71073	0.71073	0.71073
Crystal system		monoclinic	monoclinic	monoclinic	monoclinic	monoclinic
Space group		C2/c	C2/c	C2/c	C2/c	C2/c
Unit cell dimensions (Å,°):	*a*	16.2789(3)	16.210(4)	16.206(3)	16.191(4)	16.098(2)
	b	10.7307(2)	10.6863(6)	10.6965(4)	10.6521(5)	10.605(2)
	c	14.5393(3)	14.4936(9)	14.4922(5)	14.4247(6)	14.405(2)
	γ	90	90	90	90	90
	β	93.017(2)	92.95(3)	93.185(8)	93.183(9)	93.218(16)
	γ	90	90	90	90	90
Volume (Å^3)		2536.26(8)	2508(7)	2508.2(5)	2484.0(6)	2455.3(7)
Z/Z'		4/0.5	4/0.5	4/0.5	4/0.5	4/0.5
Density (g/cm^3)		1.868	1.889	1.889	1.907	1.929
Absorption coefficient (mm^{-1})		3.234	2.013	2.012	2.032	2.056
F(000)		1464.0	1464.0	1464.0	1464.0	1464.0
Crystal size (mm)		0.15·0.10·0.05	0.20·0.20·0.05	0.15·0.15·0.05	0.30·0.30·0.05	0.20·0.15·0.05
Θ-range for data collection (°)		10.88 to 152.476	7.626 to 52.826	9.138 to 54.27	9.166 to 54.536	7.41 to 53.724
Min/max indices:	h	-15/20	-3/2	-10/10	-10/10	-18/18
	k	-13/12	-12/13	-12/12	-12/12	-10/10
	l	-18/17	-18/17	-18/17	-18/17	-15/15
reflect. Collected/unique		10199/2632	4042/378	5096/823	5033/816	4921/824
R_{int}		0.0199	0.0235	0.0256	0.0250	0.0636
Data/restrains/parameters		2632/2/196	378/127/199	823/32/200	816/14/196	824/32/196
Goodness-of-fit on F_2		1.106	1.104	1.107	1.110	1.145
Final R_1/wR_2 (I>2δ1)		0.0296/0.0847	0.0210/0.0516	0.0300/0.0756	0.0272/0.0692	0.0620/0.1739
R1/ wR2 (all data)		0.0315/0.0863	0.0231/0.0537	0.0378/0.0796	0.0364/0.0742	0.0989/0.1988

Table S1 Crystallographic data of $ZnAsp_2 \cdot 1.5H_2O$ and experimental details - *continuation*

Pressure (GPa)		0.85	1.15	1.44	1.62	1.82
Formula weight		713.18	713.18	713.18	713.18	713.18
Wavelength (Å)		0.71073	0.71073	0.71073	0.71073	0.71073
Crystal system		monoclinic	monoclinic	monoclinic	monoclinic	monoclinic
Space group		*C*2/*c*	*C*2/*c*	*C*2/*c*	*C*2/*c*	*C*2/*c*
Unit cell dimensions (Å,°):	*a*	16.027(2)	16.005(3)	15.950(3)	15.916(5)	15.819(4)
	b	10.5656(8)	10.4707(8)	10.4707(8)	10.4593(12)	10.4316(10)
	c	14.3681(11)	14.2629(10)	14.263(1)	14.2566(15)	14.2229(13)
	γ	90	90	90	90	90
	β	93.12(2)	93.08(3)	93.08(3)	93.09(4)	93.03(3)
	γ	90	90	90	90	90
Volume ($Å^3$)		2429.5(4)	2386.8(5)	2378(5)	2369.9(8)	2343.8(6)
Z/Z'		4/0.5	4/0.5	4/0.5	4/0.5	4/0.5
Density (g/cm^3)		1.950	1.985	1.992	1.999	2.021
Absorption coefficient (mm^{-1})		2.078	2.115	2.122	2.130	2.154
F(000)		1464.0	1464.0	1464.0	1464.0	1464.0
Crystal size (mm)		0.20·0.15·0.05	0.20·0.15·0.05	0.20·0.15·0.05	0.20·0.15·0.05	0.20·0.15·0.05
Θ-range for data collection (°)		9.576 to 54.17	7.48 to 54.03	7.484 to 54.03	7.49 to 54.058	7.51 to 54.012
Min/max indices:	h	-3/3	-3/3	-3/3	-3/3	-3/3
	k	-13/13	-12/13	-12/13	-12/13	-12/13
	l	-18/18	-18/17	-18/17	-18/17	-17/17
reflect. Collected/unique		5840/549	4732/436	4750/437	4699/433	4678/429
R_{int}		0.0886	0.0695	0.0632	0.1007	0.0676
Data/restrains/ parameters		549/113/196	436/134/196	437/110/196	433/126/196	429/130/196
Goodness-of-fit on F^2		1.110	1.080	1.084	1.140	1.115
Final R_1/wR_2 ($I>2\delta_1$)		0.0401/0.0588	0.0314/0.0678	0.0306/0.0667	0.0392/0.0862	0.0324/0.0679
R_1/ wR_2 (all data)		0.0839/0.0715	0.0530/0.0772	0.0521/0.0760	0.0655/0.0990	0.0577/0.0811

Table S1 Crystallographic data of $ZnAsp_2 \cdot 1.5H_2O$ and experimental details - *continuation*

Pressure (GPa)		2.15	2.49	2.91	3.54	3.92
Formula weight		713.18	713.18	713.18	713.18	713.18
Wavelength (Å)		0.71073	0.71073	0.71073	0.71073	0.71073
Crystal system		monoclinic	monoclinic	monoclinic	monoclinic	monoclinic
Space group		*C*2/*c*	*C*2/*c*	*C*2/*c*	*C*2/*c*	*C*2/*c*
Unit cell dimensions (Å,°):	*a*	15.782(4)	15.731(3)	15.680(3)	15.550(3)	15.612(4)
	b	10.3838(9)	10.3513(6)	10.3060(8)	10.2262(7)	10.2330(9)
	c	14.1615(13)	14.1190(9)	14.0720(12)	13.9663(11)	13.9799(14)
	γ	90	90	90	90	90
	β	92.99(3)	92.92(2)	92.80(3)	92.62(3)	92.78(3)
	γ	90	90	90	90	90
Volume ($Å^3$)		2317.5(6)	2296.1(4)	2271.3(6)	2230.7(7)	2219(4)
Z/Z'		4/0.5	4/0.5	4/0.5	4/0.5	4/0.5
Density (g/cm^3)		2.044	2.063	2.086	2.124	2.135
Absorption coefficient (mm^{-1})		2.178	2.198	2.222	2.263	2.275
F(000)		1464.0	1464.0	1464.0	1464.0	1464.0
Crystal size (mm)		0.20·0.15·0.05	0.20·0.15·0.05	0.20·0.15·0.05	0.20·0.15·0.05	0.20·0.15·0.05
Θ-range for data collection (°)		7.54 to 53.304	7.56 to 53.474	7.584 to 53.668	7.434 to 54.052	7.448 to 54.1
Min/max indices:	h	-3/3	-3/3	-3/3	-3/3	-3/3
	k	-12/13	-12/13	-12/12	-12/12	-12/12
	l	-17/17	-17/17	-17/17	-17/17	-17/17
Reflect. Collected/unique		4622/427	4599/424	4527/413	4443/413	4400/ 407
R_{int}		0.0688	0.0690	0.0710	0.0729	0.0765
Data/restrains/ parameters		427/134/199	424/129/199	413/130/199	413/129/199	407/125/196
Goodness-of-fit on F^2		1.104	1.081	1.092	1.138	1.098
Final R_1/wR_2 ($I>2\delta_1$)		0.0343/0.0587	0.0291/0.0572	0.0317/0.0530	0.0332/0.0504	0.0333/0.0609
R_1/ wR_2 (all data)		0.0603/0.0698	0.0532/0.0668	0.0544/0.0615	0.0576/0.0574	0.0633/0.0735

Table S2 Crystallographic data of $ZnAsp_2 \cdot 2H_2O$ and experimental details.

Pressure (GPa)		0.001	0.05	0.15	0.40	0.70
Formula weight		731.19	731.19	731.19	731.19	731.19
Wavelength (Å)		1.54184	0.71073	0.71073	0.71073	0.71073
Crystal system		triclinic	triclinic	triclinic	triclinic	triclinic
Space group		$P\bar{1}$	$P\bar{1}$	$P\bar{1}$	$P\bar{1}$	$P\bar{1}$
Unit cell dimensions (Å,°):	*a*	8.7873(3)	8.7874(12)	8.7725(11)	8.7385(18)	8.687(4)
	b	9.5061(3)	9.4894(15)	9.4694(14)	9.419(3)	9.359(3)
	c	9.8114(3)	9.812(3)	9.803(3)	9.773(6)	9.7666(19)
	α	111.784(3)	111.913(19)	111.906(19)	111.86(4)	111.78(3)
	β	105.625(3)	105.549(16)	105.503(16)	105.42(3)	105.20(3)
	γ	107.721(3)	107.728(13)	107.777(12)	107.88(2)	108.04(4)
Volume (Å^3)		653.73(4)	652.1(2)	649.0(2)	641.4(5)	633.9(4)
Z/Z’		1/0.5	1/0.5	1/0.5	1/0.5	1/0.5
Density (g/cm^3)		1.857	1.862	1.871	1.893	1.915
Absorption coefficient (mm^{-1})		3.183	1.940	1.949	1.973	1.996
F(000)		376.0	376.0	376.0	376.0	376.0
Crystal size (mm)		0.25·0.10·0.02	0.25·0.20·0.05	0.25·0.20·0.05	0.25·0.20·0.05	0.25·0.20·0.05
Θ-range for data collection (°)		10.768 to 152.508	7.576 to 54.68	7.596 to 54.348	7.64 to 54.638	7.702 to 54.98
Min/max indices:	h	-11/9	-11/10	-10/10	-11/10	-11/10
	k	-11/11	-11/12	-11/12	-11/12	-11/12
	l	-12/12	-7/7	-7/7	-7/7	-7/7
Reflect. Collected/unique		13265/2706	2655/479	2630/474	2596/475	2494/470
R_{int}		0.0199	0.0521	0.0684	0.0647	0.0624
Data/restrains/ parameters		2706/0/199	479/132/198	474/132/198	475/131/198	470/131/198
Goodness-of-fit on F^2		1.089	1.120	1.126	1.102	1.100
Final R_1/wR_2 ($I>2\delta_1$)		0.0240/0.0649	0.0461/0.1208	0.0515/0.1102	0.0399/0.0905	0.0398/0.0903
R_1/ wR_2 (all data)		0.0242/0.0651	0.0621/0.1347	0.0693/0.1287	0.0570/0.1023	0.0569/0.1021

Table S2 Crystallographic data of $ZnAsp_2·2H_2O$ and experimental details - *continuation*

Pressure (GPa)		1.02	1.54	2.53	3.05	3.53	4.02
Formula weight		731.19	731.19	731.19	731.19	731.19	731.19
Wavelength (Å)		0.71073	0.71073	0.71073	0.71073	0.71073	0.71073
Crystal system		triclinic	triclinic	triclinic	triclinic	triclinic	triclinic
Space group		$P\bar{1}$	$P\bar{1}$	$P\bar{1}$	$P\bar{1}$	$P\bar{1}$	$P\bar{1}$
Unit cell dimensions (Å,°):	*a*	8.668(4)	8.594(4)	8.484(3)	8.415(4)	8.386(5)	8.343(7)
	b	9.308(4)	9.235(3)	9.110(3)	9.033(4)	8.983(3)	8.947(6)
	c	9.738(3)	9.6951(16)	9.6213(14)	9.5778(17)	9.5498(19)	9.537(3)
	α	111.52(3)	111.39(2)	110.91(2)	110.49(3)	110.18(2)	110.02(4)
	β	105.23(3)	104.94(2)	104.75(2)	104.77(3)	104.73(3)	104.76(4)
	γ	108.10(4)	108.42(3)	108.76(3)	108.92(5)	109.15(4)	109.16(7)
Volume ($Å^3$)		628.5(5)	616.2(4)	597.6(3)	586.8(5)	580.5(5)	575.2(7)
Z/Z'		1/0.5	1/0.5	1/0.5	1/0.5	1/0.5	1/0.5
Density (g/cm^3)		1.932	1.971	2.032	2.069	2.092	2.111
Absorption coefficient (mm^{-1})		2.013	2.053	2.117	2.156	2.180	2.200
F(000)		376.0	376.0	376.0	376.0	376.0	376.0
Crystal size (mm)		0.25·0.20·0.05	0.25·0.20·0.05	0.25·0.20·0.05	0.25·0.20·0.05	0.25·0.20·0.05	0.25·0.20·0.05
Θ-range for data collection (°)		7.736 to 54.144	7.83 to 53.388	7.968 to 54.224	8.05 to 54.802	8.11 to 54.732	8.148 to 54.618
Min/max indices:	h	-4/4	-4/4	-4/4	-4/4	-5/4	-5/5
	k	-9/9	-9/9	-9/8	-8/8	-8/8	-7/7
	l	-12/12	-12/12	-12/12	-12/12	-12/11	-12/11
Reflect. Collected/unique		2540/461	2559/458	2469/444	2377/440	2344/421	2352/430
R_{int}		0.1072	0.1148	0.1056	0.0645	0.0842	0.1240
Data/restrains/ parameters		461/139/199	458/136/198	444/134/198	440/126/198	421/126/198	430/134/204
Goodness-of-fit on F^2		1.107	1.120	1.057	1.071	1.137	1.149
Final R_1/wR_2 ($I>2\delta_1$)		0.0534/0.1048	0.0491/0.1078	0.0514/0.1124	0.0360/0.0736	0.0536/0.1339	0.0519/0.1136
R_1/ wR_2 (all data)		0.0913/0.1382	0.0874/0.1420	0.0847/0.1409	0.0665/0.0850	0.0952/0.1625	0.1168/0.1560

S2. Figures

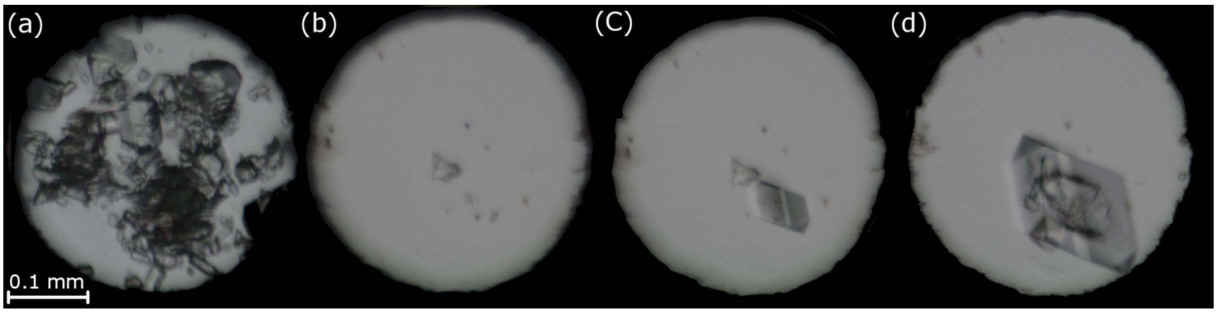

Figure S1 Stages of $ZnAsp_2 \cdot 1.5H_2O$ growth in isochoric conditions in the diamond-anvil cell chamber: (a) spontaneous crystallization of powder at 433 K; (b) one seed at 423 K; (c) 353 K; and (d) 0.29 GPa/296 K. The ruby chips for pressure calibration lie by the middle of the hole.

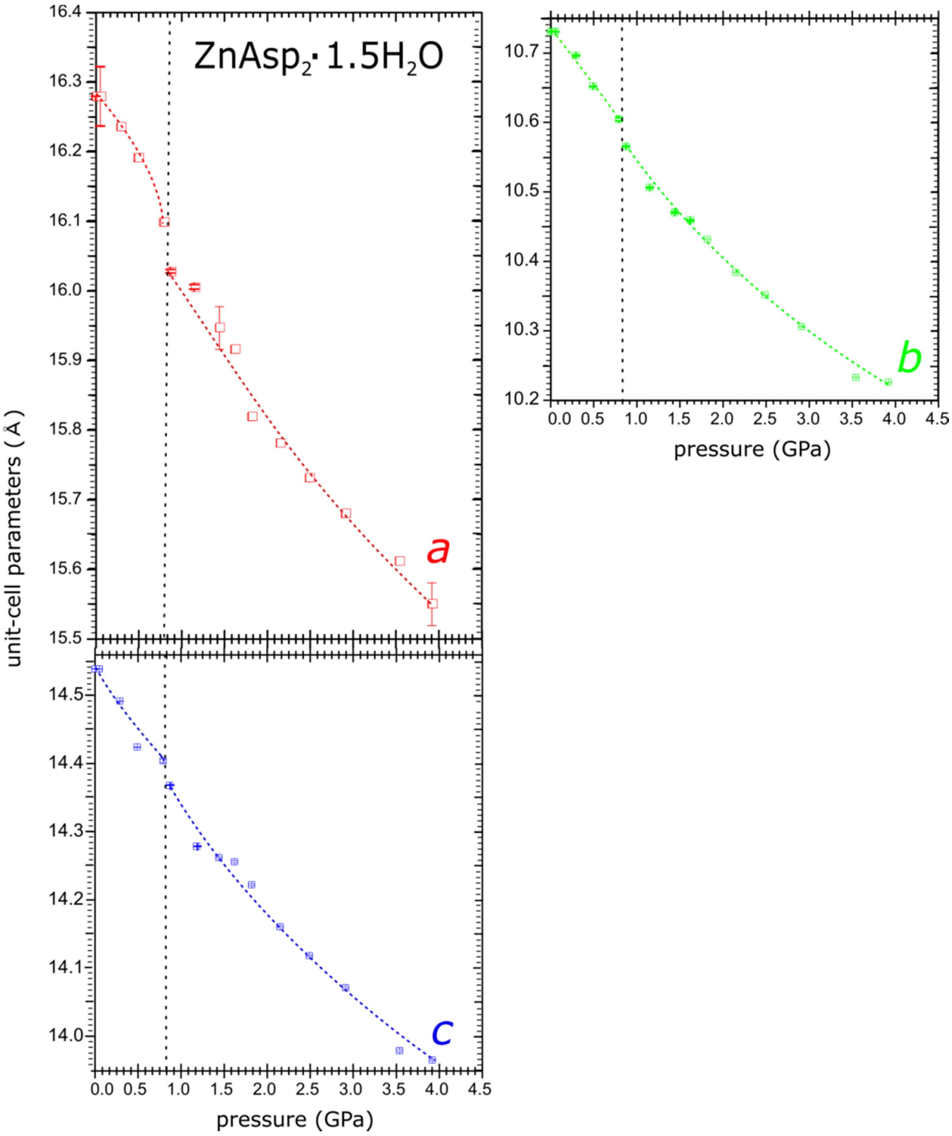

Figure S2 The unit-cell parameters of $ZnAsp_2 \cdot 1.5H_2O$, plotted as a function of pressure. The vertical dashed line indicates the phase transition.

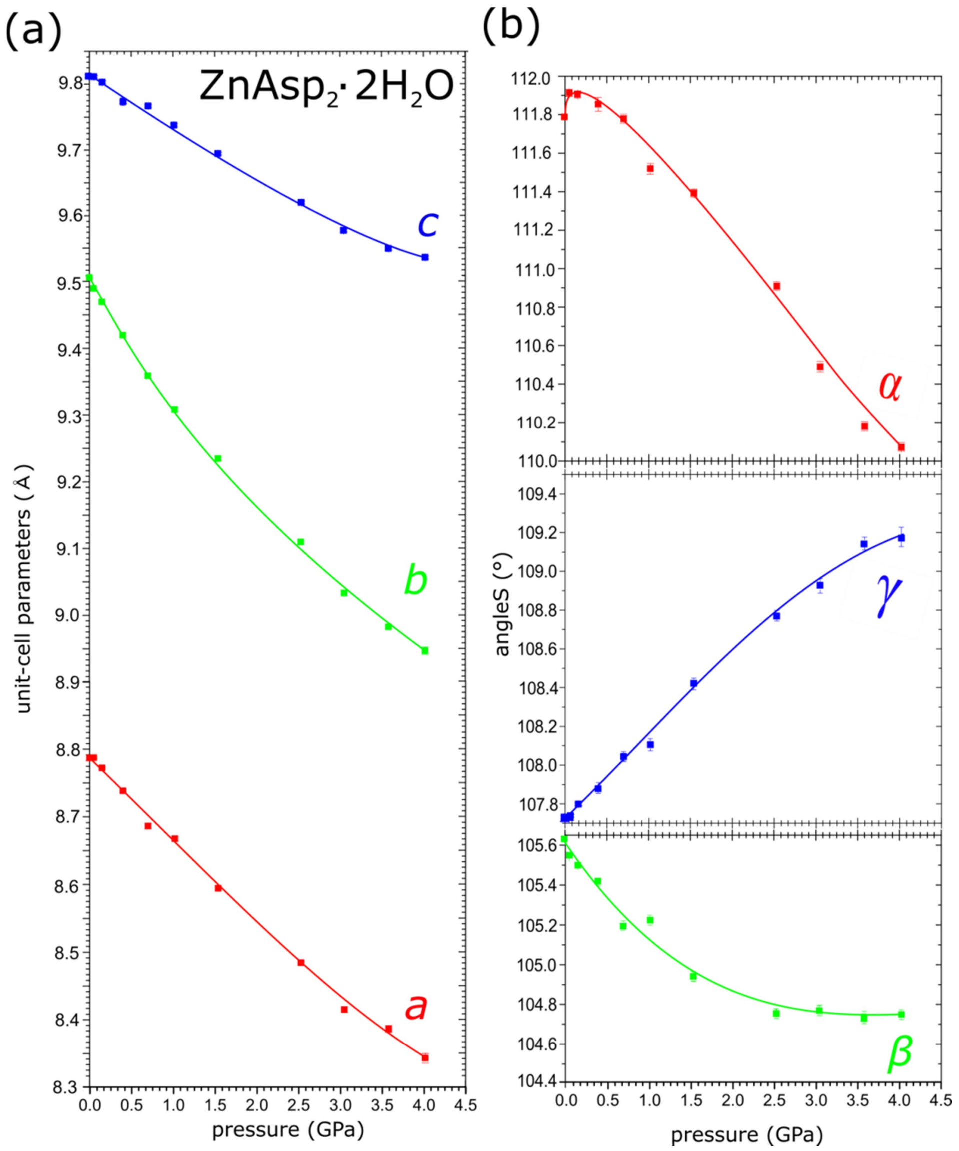

Figure S3 The unit-cell parameters and α, β and γ angles of $ZnAsp_2{\cdot}2H_2O$, plotted as a function of pressure.

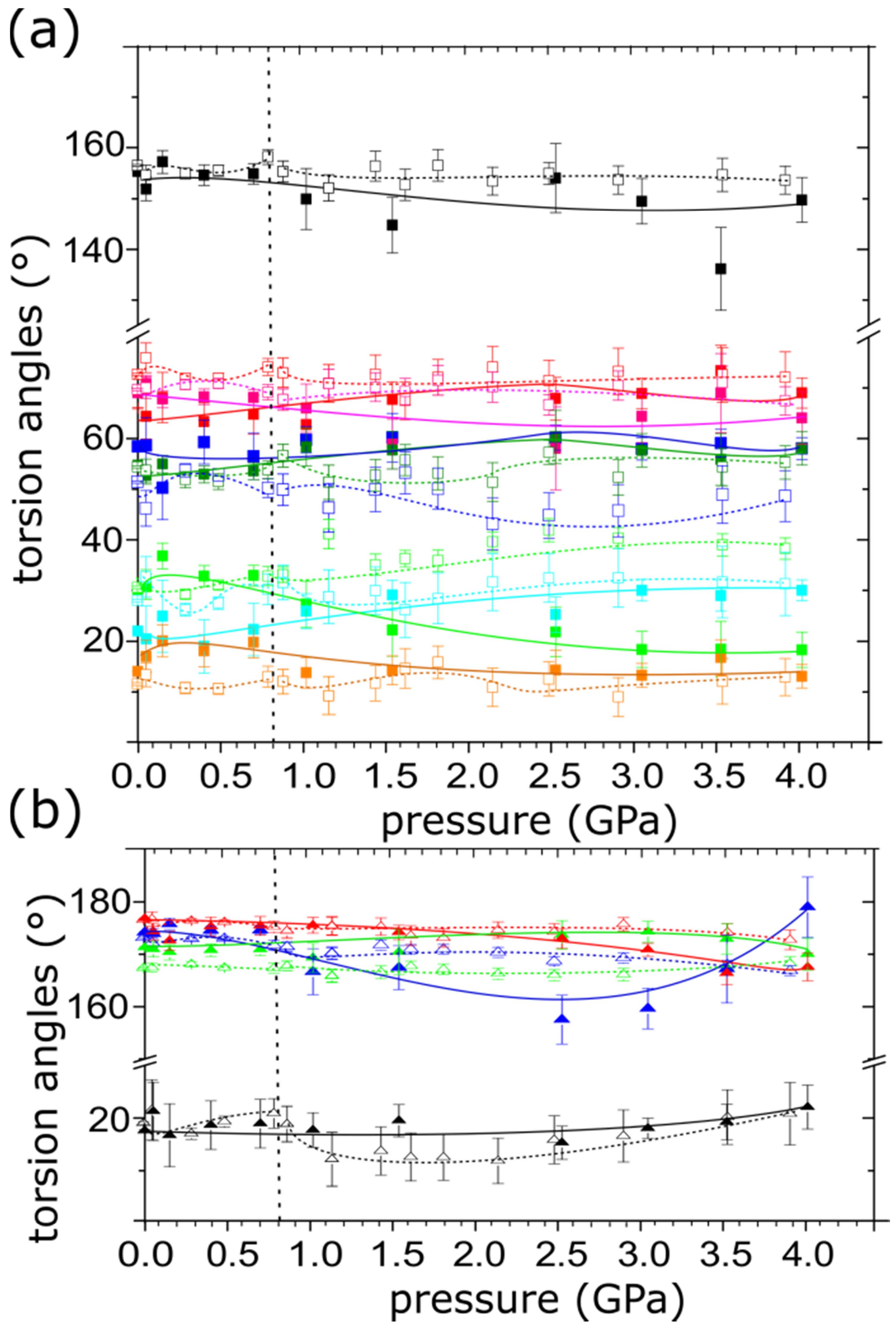

Figure S4 (a) Torsion angles in aspartate anions: C1-C2-C3-N3 (red); C1-C2-C3-C4 (blue); N3-C3-C4-O3 (light green); O2-C1-C2-C3 (cyan); C11-C12-C13-N13 (pink); C11-C12-C13-C14 (green); N13-C13-C14-O13 (orange) and O12-C11-C12-C13 (black); (b) torsion angles involving Zn-O coordination bonds: Zn1-O1-C1-C2 (black); Zn1-O11-C11-C12 (blue); Zn1-O4-C4-C3 (green); Zn1-O14-C14-C13 (red). Full symbols/lines for $ZnAsp_2 \cdot 2H_2O$, empty symbols/dotted lines for $ZnAsp_2 \cdot 1.5H_2O$.

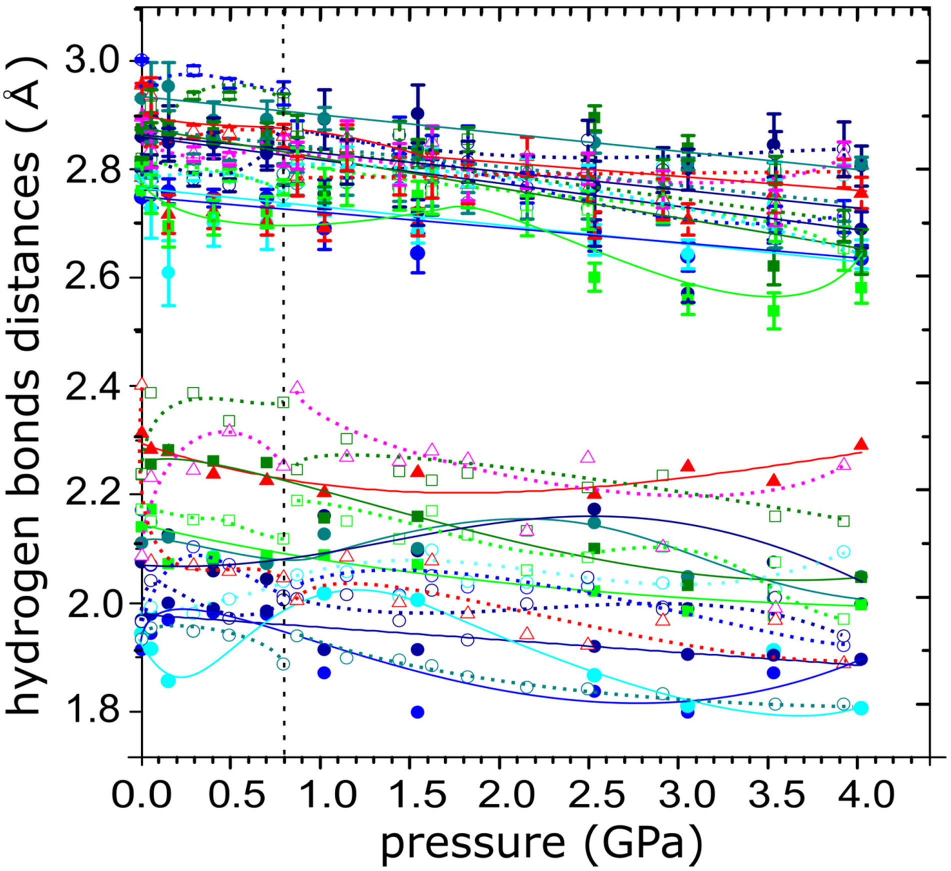

Figure S5 Intramolecular hydrogen bonds N13H···O11 and N3H···O2 (triangles); H2O-mediated H-bonds: O1wH···O14, O1wH···O2, O2wH···O12, N3H···O1w and O2wH···O1w (circles) and two other H-bonds: N13H···O2; N3H···O13 (squares) in $ZnAsp_2 \cdot 1.5H_2O$ (empty symbols) and $ZnAsp_2 \cdot 2H_2O$ (full symbols).

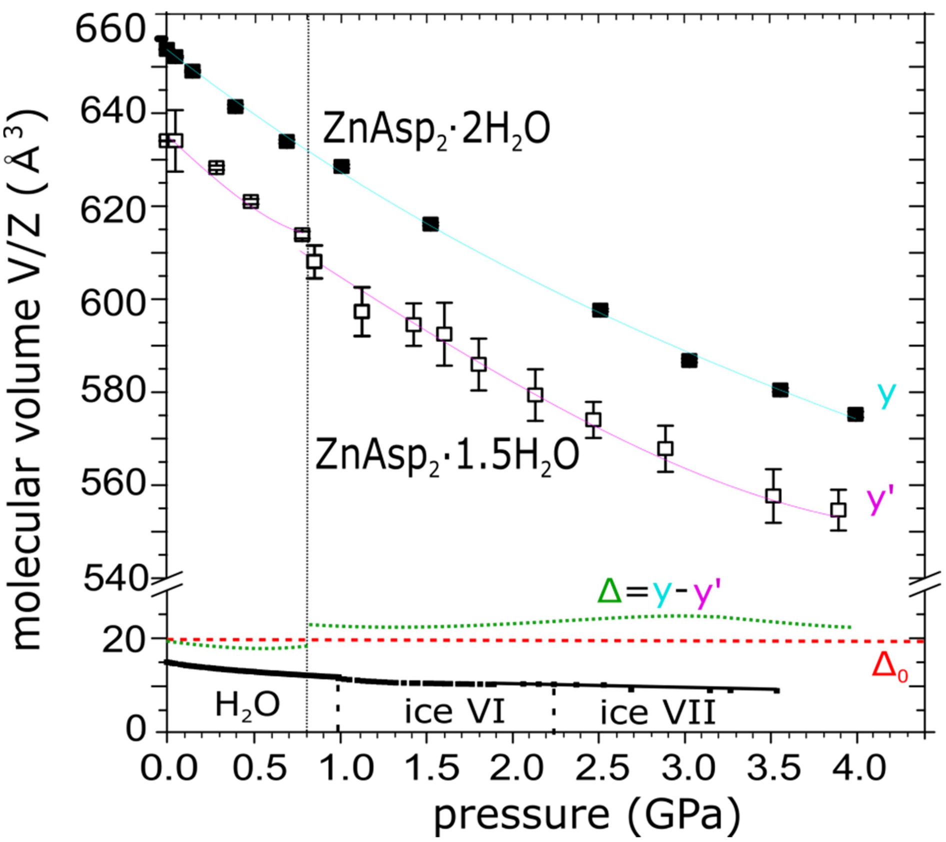

Figure S6 The molecular volume (V/Z) of $ZnAsp_2 \cdot 2H_2O$ (full symbols) and $ZnAsp_2 \cdot 1.5H_2O$ (open symbols) plotted as a function of pressure. The molecular volume of water (black line; *i.e.* one H_2O molecule in liquid and ices VI and VII) as a function of pressure compare to the difference of the molecular volume (V_m=V/Z) of $ZnAsp_2 \cdot 2H_2O$ and that of $ZnAsp_2 \cdot 1.5H_2O$ (Δ, green line); dependence of the Δ_0 is this difference at 0.1 MPa.

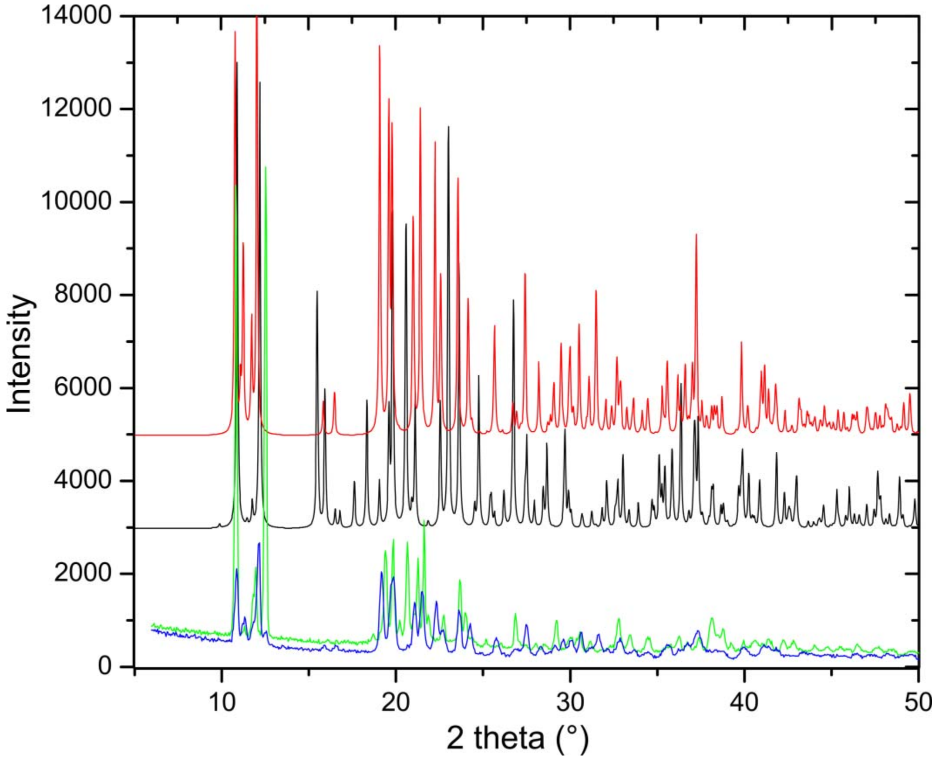

Figure S7 X-Ray powder diffraction (XRPD) of $ZnAsp_2 \cdot 2H_2O$ (black line) and $ZnAsp_2 \cdot 1.5H_2O$ (red); the $ZnAsp_2 \cdot 2H_2O$ sample after being kept at 170°C for 1 hour (geen); and the same sample after keeping in the 100% humid air at 50°C for 24 hours (blue).

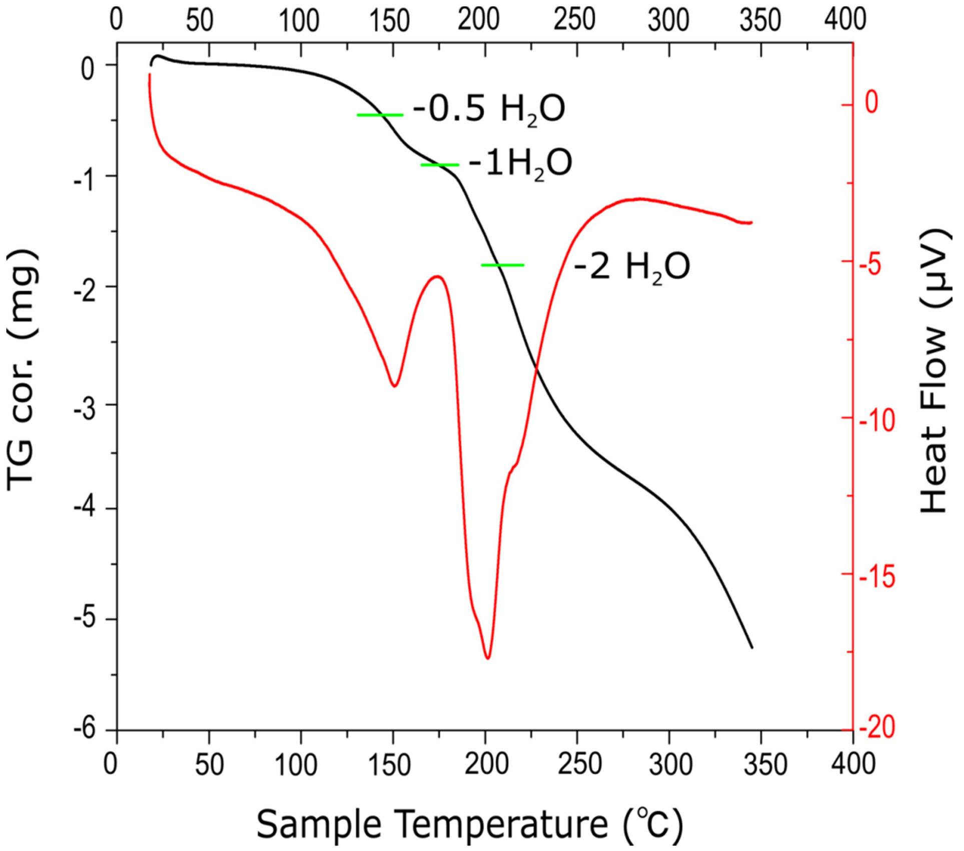

Figure S8 TGA and DSC analysis of $ZnAsp_2{\cdot}2H_2O$. Sample 18,2 mg, in the N2 atmosphere on an Instrument Setsys 1200 Setaram, between 293 K and 573 K, with the scan speed of 5 K/min.

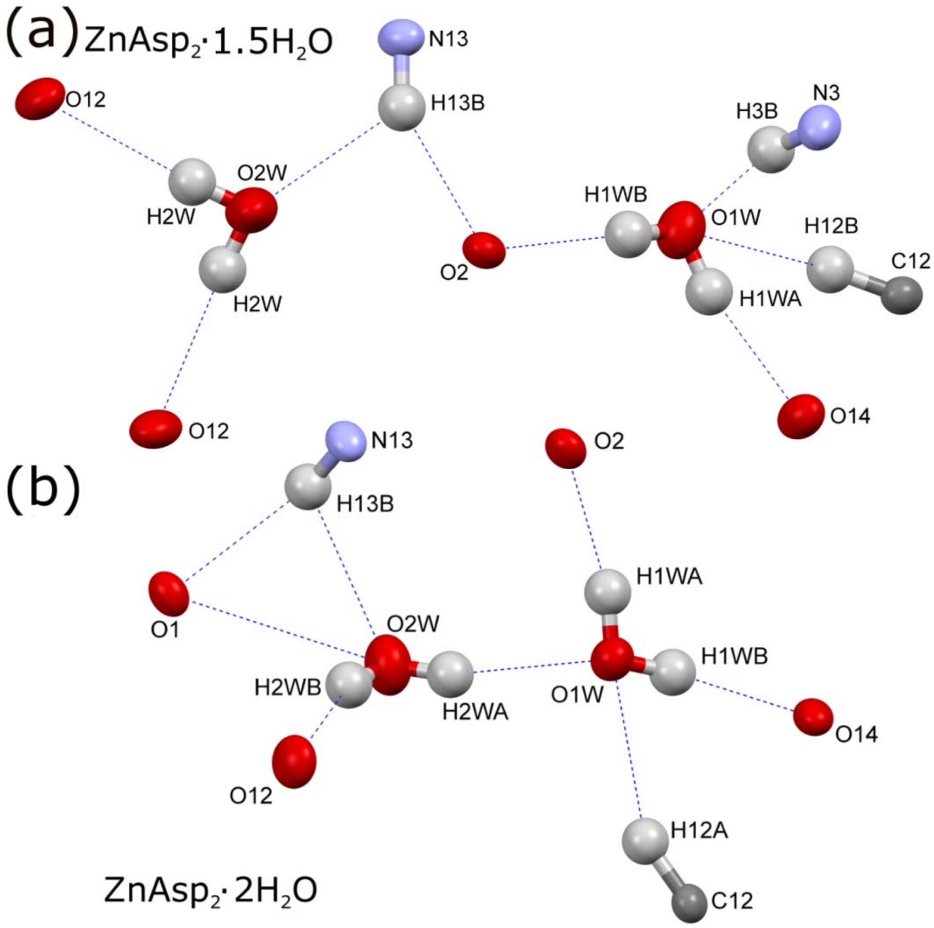

Figure S9 The H-bonding patterns of water molecules in the structures of: (a) ZnAsp2·1.5H2O; and (b) ZnAsp2·2H20.

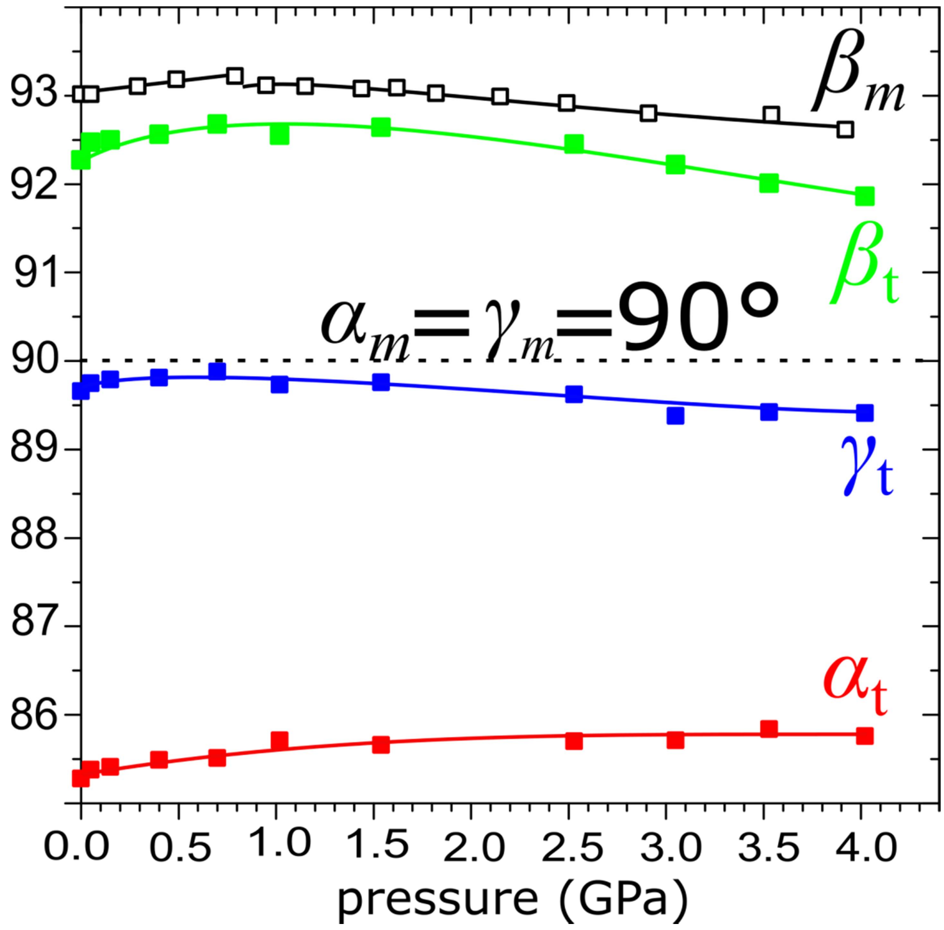

Figure S10 The angular dimensions of the pseudo(monoclinic) lattice C of ZnAsp2·2H2O (full symbols and lines) and ZnAsp2·1.5H20 (open symbols and dashed lines) as a function of pressure.
